# Evaluating an Automated Number Series Item Generator Using Linear Logistic Test Models

**DOI:** 10.3390/jintelligence6020020

**Published:** 2018-04-02

**Authors:** Bao Sheng Loe, Luning Sun, Filip Simonfy, Philipp Doebler

**Affiliations:** 1 The Psychometrics Centre, Cambridge Judge Business School, University of Cambridge, Trumpington Street, Cambridge CB2 1AG, UK; ls523@cam.ac.uk; 2 Department of Psychology, University of East Anglia, Norwich NR4 7TJ, UK; filipsimonfy@gmail.com; 3 Faculty of Statistics, TU Dortmund University, 44227 Dortmund, Germany; doebler@statistik.tu-dortmund.de

**Keywords:** cognitive models, automatic item generation, number series, Rasch model, Linear Logistic Test Models

## Abstract

This study investigates the item properties of a newly developed Automatic Number Series Item Generator (ANSIG). The foundation of the ANSIG is based on five hypothesised cognitive operators. Thirteen item models were developed using the numGen R package and eleven were evaluated in this study. The 16-item ICAR (International Cognitive Ability Resource^1^) short form ability test was used to evaluate construct validity. The Rasch Model and two Linear Logistic Test Model(s) (LLTM) were employed to estimate and predict the item parameters. Results indicate that a single factor determines the performance on tests composed of items generated by the ANSIG. Under the LLTM approach, all the cognitive operators were significant predictors of item difficulty. Moderate to high correlations were evident between the number series items and the ICAR test scores, with high correlation found for the ICAR Letter-Numeric-Series type items, suggesting adequate nomothetic span. Extended cognitive research is, nevertheless, essential for the automatic generation of an item pool with predictable psychometric properties.

## 1. Introduction

### 1.1. Automatic Item Generation

Automatic Item Generation (AIG) is an emerging research area in which quantitative methods such as psychometrics and cognitive psychology are integrated to assist in test development using computational algorithms [1,2]. AIG can be characterised as the process of using models to generate items that are statistically pre-calibrated [3]. These items are derived from an item model which is an operationalised representation of the latent ability or construct to be measured. The core of the item model consists of elements that can be systematically manipulated in order to generate a diversified item bank [4]. As a result, an affluence of novel and statistically pre-calibrated items can potentially be created from a single item model [3].

Significant advancement in research and practice has led to AIGs being successfully used in research of intelligence, educational and medical measurement [3]. For example, within intelligence research, AIGs have been employed to create items in diverse content areas including, but not limited to, mental rotation [5], spatial and abstract reasoning [6,7,8], figural matrices [9,10], anagram problems [11], Latin square tasks [12] and quantitative reasoning items [13].

### 1.2. Inductive Reasoning

Intelligence research often employs tests of inductive reasoning which is considered a marker of ‘fluid intelligence’ [14,15]. According to [16,17], it is central to the concept of intelligence testing. Inductive reasoning involves generating and testing hypotheses and generalisation of findings from a set of predefined instances [18]. Similarly, Spearman [10] defined inductive reasoning as a process consisting of perception of units, logical induction of correspondences between these units and application of the identified rules to other units. It is typically measured by series completion tasks, which form a common part of many standardised intelligence test batteries [19,20,21].

Series completion tasks are usually based on schemes which define the succession and enable identification of consecutive elements. Such schemes were first introduced by Simon and Kotovsky [22], who developed the theory and descriptive language applied to letter series problems. Holzman, Pellegrino and Glaser [23] extended their classical scheme to incorporate number series and further emphasised the cognitive mechanisms rather than the problem features of the series. They proposed that the number series completion task should be based on (1) relations detection, (2) discovery of periodicity, (3) pattern description and (4) extrapolation.

Identification of relation detection is based on respondents scanning the number series and postulating hypotheses about the relationships between the elements. Research indicates that the difficulty of detecting a relationship in the number series task is dependent on the arithmetic operations involved [23] and the initial practice that participants had with structural relations [24]. However, it is not limited to these reasons as a source of explanation. The emphasis is about hypothesising how the elements in the series are related to one another. Discovery of periodicity is the ability to detect the elements which constitute one complete cycle of the pattern. The relationship of the elements is based on adjacent positions until proved otherwise. At this stage, either the participants assume that a new period is introduced, or they have identified an incorrect pattern of cycle. The number series task is conceived to be harder if the participant’s pattern prediction is wrong [25]. Pattern description identifies the relationship governing the remaining positions within the period and defines the rule for the sequence which is then extrapolated to its missing elements [23]. Participants are able to integrate the previously formed hypotheses to deduce the rules that govern the number series and extrapolate the rules to complete the number series by filling in the missing elements [26]. Extrapolation is the means by which the numbers are generated to complete the sequence based on the previous three components.

By definition, the three cognitive determinants proposed by Holzman et al. [23] are necessary in order to solve a series completion task. However, there is little systematic guidance regarding the development of new number series item models. Arendasy and Sommer [26] proposed four generative components for number series, that is, rule span, number of rules, rule complexity and periodicity. These four rules focused more on the problem features and did not necessarily represent or explain how participants use different cognitive domains to solve the number series problems. Moreover, individual differences in working-memory capacity and overall performance on the number series were found only with children and not with adults [23]. While these findings provide a preliminary understanding of the performance of series completion tasks, they are inadequate in providing a more detailed account of the cognitive processes involved in solving the number series items in the adult population.

### 1.3. Automatic Number Series Item Generator

The creation of an Automatic Number Series Item Generator (ANSIG), based on the four cognitive determinants proposed by Holzman et al. [23], has several important advantages. First, systematic design of number series items will allow researchers to carefully manipulate important item parameters which may contribute to the performance of the tasks. Second, a clear and detailed account of item difficulty variance may allow researchers to better understand how individual differences play a role in number series tests. Third, in longitudinal assessment of cognitive functioning studies, researchers may employ item variants rather than the same set of items across different time points in order to evaluate cognitive ability, reducing the possibility of practice effect and minimising item exposure [27,28]. Fourth, in cases where adaptive testing is not feasible as part of a larger suite of psychological test battery, using efficient methods such as genetic algorithm for item selection strategy can be adopted to select a subset of items from an item bank created by the AIG [29]. Fifth, the administration of an on-the-fly, adaptive test with calibrated item models serves as an alternative cost-effective approach to testing [30].

Empirical information to build a complete cognitive model is often unavailable during the test design phase. While schemas provided by the subject matter experts may be a good starting point, these predictions of item difficulty are often inaccurate [31,32]. Therefore, this study employs the model-based (or strong-theory) approach [33] in which the manipulation of the item features is based on a model of hypothesised cognitive operators. Following this approach enables the development of items with psychometric attributes to be theoretically predictable.

In this study, we propose five cognitive operators (i.e., the rules) which individuals (adults) may employ to solve the number series items within the item model: (1) apprehension of succession (AOS), which detects a single coherent number series without involving any arithmetic operation; (2) identification of parallel sequences (PS), where two parallel number series are present; (3) object cluster formation (CF), which identifies clusters of numbers embedded in the series; (4) identification of non-progressive coefficient patterns (NPCP), where the missing value is determined solely by the preceding elements; and (5) identification of complex progressive relationships with a change in the coefficients (PCP), which requires identification of the pattern in the increments of the consecutive values. Example items are shown in Table 1. Individuals use at least one of these 5 cognitive operators to reason logically and identify the correct answer(s) in the number series task.

These five cognitive operators map onto the four determinants proposed by Holzman et al. [23] with variant levels of weights. As indicated in Table 1, the first cognitive operator, apprehension of succession serves as a baseline. Building on this, items with parallel sequences poses more processing demand on the discovery of periodicity, as respondents need to realise the existence of two rather than one series in the item, through detecting inconsistent patterns between conjunctive numbers. Identifying object clusters formation within the number series requires a high level of relation detection, because it requires respondents to identify groups of elements that are linearly sequential rather than a single element establishing a (parallel) relation with the adjacent element. Respondents, therefore, must form a hypothesis based on the relations of the elements and the discovery a cycle of pattern until an element contradicts this presumption [23,34]. At this point, respondents either begin forming new hypothesis or have miscalculated. In the next phase, respondents integrate their hypothesis with the completion of pattern description to deduce the rules that govern the number series. The inferred rules are then extrapolated to find the missing element(s) of the number series. The last two cognitive operators adopt the identification of simple and complex arithmetic operations respectively, exhibiting increasing weights on the pattern description and extrapolation. Therefore, the increased dependency on the processing stages of the cognitive determinants in relation to the five cognitive variables lead to varying levels of impact on item difficulty.

### 1.4. ANSIG Item Models

Thirteen item models were designed based on a non-arbitrary combination of the 5 cognitive operators. The generation of the items from each model is an integration of the item cloning approach and the item attribute approach [35]. Following the item cloning procedure described by Glas and van der Linden [36], attributes from the parent item can be filled in by specified substitution sets [37,38]. In this study, the numeric values in the number series are the substitution sets within each item family, allowing the ability to clone large number of items while maintaining the same item templates. Simple mathematical arithmetic operators were involved in the generation of the items but were not considered substantial to impact difficulty levels significantly and were therefore, not treated as substitution sets. Furthermore, based on the attribute approach proposed by Freund, Hofer and Holling [39] and Holling, Bertling and Zeuch [40], the structural variants of the item templates are created based on elementary operators [41], which in this case, is the combination of the five cognitive operators.

A description of the item models can be found in Table 2. A design matrix (Q-matrix) was created to account for these five cognitive operators. Following a theoretical cognitive model reduces the chances of having to revise our proposed item logic. Furthermore, using fixed item logic extends our understanding of how the cognitive mechanisms affect the item model difficulty. Therefore, the rules employed to develop an item model were not merely operational in its workings but rather designed to investigate individual differences in inductive reasoning.

The item difficulty of each item model is largely dependent on the combination of cognitive operations that individuals employ while trying to solve the problems. Carpenter, Just & Shell [42] argued that the most difficult problems in Raven Progressive Matrices are those that place a heavy burden on the working memory. These authors proposed that the amount of information, which one could maintain in the working memory, is an important indicator of the individual’s reasoning ability. Furthermore, they suggested that performance is not only based on the number of rules that are required to solve each problem but are also attributable to differences in the way one utilises the rules. Their argument was supported through a thorough item analysis of the Raven Progressive Matrices. Difficult items are those that require a large number of rules held in the working memory and that require the use of more difficult rules in order to solve the items [42]. Babcock [43] also supported the notion that the difficulty of Raven Matrices is due to the processing resources in the working memory available to the participants. Therefore, in accordance with the theory of individual differences in working memory, we propose that items from different item models may exhibit different item difficulty levels, as the cognitive operators underlying the item models require variable amount of working memory and involve different mental processes. Moreover, the item difficulty in each item model increases when individuals employ more cognitive operators to solve an item. This is due to a heavy burden in the information processing resources when more than one cognitive operators are held in the working memory when the individual is attempting to solve the item. Thus, item models designed to involve more cognitive operators will be more difficult than those that use fewer cognitive operators.

### 1.5. numGen R Package

It may be of interest to readers to understand how the number series generator was created. Hence, a written description of the numGen R package item generator is provided in this section. The numGen R package [44] was developed based on the proposed cognitive operators, which led to the creation of 13 R functions corresponding to each item model. Each function can generate many items that can be considered to be an item clone of one another. Constraints were not employed in the generator to allow more flexibility in the item generation.

Item model 1 is based on the first operator only. The starting value of the number sequence could begin from any value, while the following values are of ordered constant of 1, 10 or 100 (e.g., 1 2 3 4 (5)). The sequence length is fixed at 5. Given a fixed sequence length, a starting value and a specific constant, a large number of items could be generated.

Item model 2 is designed based on the third cognitive operator only, which is the understanding of object clusters. The order of sequential numbers is not important but it is essential to recognise that the sequence of values is clustered in groups. Thus, it is critical that there is an equal length of repetitive numbers in each group. For example, in the number series 1 1 1 23 23 (23), there are 2 clustered groups. The first being ‘1’, while the second being ‘23’. Each of this value is repeated three times, with a fixed length of 6 sequences. Thus, random numbers could be used as a representation of the clustered groups.

Item model 3 is designed based on the first and the fourth cognitive operator. The values of the numbers increase sequentially based on a mathematical operator performing on the previous value. Unlike the first item model, the increasing values are not constant. Hence, basic mathematical operators such as addition, subtraction, multiplication and division could be used to increase the order of the values. A large number of similar number sequence type questions could be generated (e.g., 1 2 4 8 16 (32)) given a starting value, a fixed length, an arithmetic operator and a numeric value.

Item model 4 is designed based on the second cognitive operator only. The logic consists of an alternating pattern of sub-sequences. Two individual sequences could first be generated: 1 2 3 4 (5) and 2 4 6 8 (10); then both sequences are combined by alternating the input of the two sequences one at a time, resulting in a final sequence of 1 2 2 4 3 6 4 8 (5) (10). In order to compute a large number of items based on item model 4, random sequences could first be generated before combining the sequences into one.

Item model 5 is designed based on the second and fourth cognitive operators. One of the two sequences is first designed based on the second cognitive operator (item model 4), before combining them to form the parallel sub-sequences within a sequence. Furthermore, there is no constraint on the pattern of the second sequence. Therefore, in practice, this could be any form of number sequence pattern.

Item models 6 and 7 are created following the theoretical models of the first and the fifth cognitive operator. The creation of this sequence requires a higher level of abstraction between the paired numbers because the abstract value that results in the change of the preceding number to the next number is not invariant. For example, in the sequence 1 2 4 7 (11), the abstract value increases in the value by 1 between each paired number. In other words, the difference between 1 and 2 is 1, while the difference between 2 and 4 is 2, so on and so forth until the desired fixed length is reached. In the above example, we showed that there is an increasing value of 1 regardless of the starting abstract value but that value need not be fixed to 1. For item model 7, an item would be 3 10 24 52 108 (220), where the abstract value begins from 7 and changes by a value of 3 instead of 1. The change in the abstract value can be randomly generated using different arithmetic operators. The R function that generates items following Item model 7 is restricted to only the use of addition and multiplication operator to calculate the abstract value in the R package. Thus, the main difference between the two item models is a change in the abstract value which consequently changes the value between the preceding and the following number in the sequence.

Item model 8 is a combination of the third and fourth cognitive operators. First, the generation of these items is based on grouping the numbers into pairs or triads (third cognitive operator), with each group not necessarily having a relationship with the preceding group of numbers. However, each group needs to have the same set of numbers. For example, in the sequence 2 5 8 11 89 (92), there are 3 groups of paired numbers. The difference between the paired numbers in each group is the same (3), adhering to the fourth cognitive model but there is no relationship between each pair of numbers.

Item models 9 and 11 follows the theoretical model of the first, third and fourth cognitive operators. An example of item model 9 is 1 1 2 3 5 8 (13), where the value of a number in the sequence is based on the addition (fourth cognitive operator) of the two preceding numbers (first and third cognitive operators). This way of generating items follow the Fibonacci sequence approach. An example of item model 11 is the sequence 1 7 14 20 40 46 (92) (98), the items are first grouped in pairs (1–7) (14–20) (40–46) (92–98), with a difference of 6 in each pair of numbers (third and fourth cognitive operator). The starting value of the first number in the pair is the multiplication of the second number in the preceding pair by 2 (first cognitive operator).

The design of item model 10 is a combination of the second and fifth cognitive operators. For example, the sequence 2 15 4 17 7 19 11 21 16 (23) (22) can be broken up into two individual sequences 2 4 7 11 16 (22) and 15 17 19 21 (23) (second cognitive operator). The logic of the fifth cognitive operator is applied to at least one of the sequences, where the next value progressively evolves from the preceding value.

Finally, item model 12 and item model 13 is a combination of the second, third and fourth cognitive operators. An instance of item model 12 is as follows: 1 22 44 2 66 88 3 (110) (132). Parallel sequencing (second cognitive operator) follows between each pair of numbers in the item, where 1 2 (3) is a sequence and (22 44) (66 88) (110) (132) is another sequence of paired numbers (third cognitive operator). The difference between the pair of numbers in each group is 22 and the difference between the second paired number of the preceding group and the first paired number of the next group is 22. The value (i.e., 22) that differentiates within the pair of numbers and between the paired groups do not need to be the same, allowing more items to be generated. For item model 13, the sequence is similar to item model 12 (e.g., 1 5 8 3 209 212 5 41 (44) (7)), with the difference being that the pair of numbers within the sequence (5 8) (209 212) (41 44) are not connected to one another.

It is possible that some item models (e.g., item models 3 and 8) were given the flexibility to change certain parameters (e.g., arithmetic operators) in the R package. However, this flexibility is part of the functional design of the generator as it is not yet intended to be used in an automated item banking system. The items in this research were, therefore, selected from a larger pool of generated items to ensure that the group of items from each item model were logically similar but appeared distinct from one another. The four arithmetic operators were not systematically controlled to generate the items and thus, mathematically ability was not included as a cognitive operator. Given the first iteration of the generator, constraints were not introduced and should thus, be considered as a separate component of the generator. Please refer to the technical manual for more details regarding the numGen R package.

### 1.6. Statistically Modelling ANSIG

Several complex statistical models have been proposed for the study of items created by automatic item generators. For example, Glas and van der Linden [45] proposed a Bayesian hierarchical model to analyses item families with multiple choice questions. It was then extended by Sinharay, Johnson and Williamson [46], which defines the family response function as an approach to summarizing the probability of a correct response given than an item is randomly generated from an item family. However, these approaches only accounts for item cloning effects in the model. Geerlings, Glas and van der Linden [47] proposed a hierarchical IRT model that not only accounts for item-cloning effects but also for item-generation rules that have fixed effects on the item difficulties. The aim was to provide higher-level explanatory variables that account for both difficulty and discrimination effects. More recently, Cho et al. [35] proposed a model for additive multilevel item structure with item attributes for each level of the item structure, extending the two-parameter logistic (2PL) item response model [48] and allowing both explanation and item generation with a multilevel item structure.

However, the Rasch model, linear logistic test model (LLTM, [49]) and the LLTM plus error [50] were instead used in this study. These models are relatively simple compared to those models mentioned in the previous paragraph. The advantage of using simpler models, which are preferred over the complex models, is that it provides a clearer understanding of how the hypothesised cognitive determinants influence the solution process of the individual and thereby, impact the difficulty of the items. The evaluation of the cognitive determinant effects in this study will thus, allow us to make an informed decision as to which complex model is more suited to be used for number series items that are automatically generated.

### 1.7. Construct Validity

An important aspect of AIG development is construct validity. Whitely [51] proposed two aspects of construct validation that stem from different types of supporting research, namely, construct representation and nomothetic span. Construct representation is concerned with recognising the theoretical concepts that influence item responses and is mostly related to the processes, strategies and knowledge structures that are involved in solving the item [51]. That is, elements within the items are manipulated to vary cognitive demands. Mathematical modelling of item difficulty has often been a chief approach in understanding construct representation because it can be used to identify cognitive processes, develop item models and empirically evaluate generated items [52]. Thus, item difficulty indirectly affects the construct representation through the cognitive processing ability of the individual. Individuals may engage in various processes simultaneously if the tasks are deemed complex. Often, the processes that are most important within a set of items determine which dimension is being measured. Even though the item features vary, the parameters of the items should vary in a predictable fashion depending on the patterns of cognitive complexity [1]. Thus, the proposed cognitive model should be accountable for all significant sources of variance in item difficulty.

Nomothetic span is concerned with the external correlates of test scores to other related measures [51]. The focus is on assessing the utility of the test for measuring individual differences. Individual differences on a test should have frequent and strong correlations with other related tests measuring the same construct. It is not unusual to have a well-supported, constructed representation but relatively low correlations with other measures [53]. In contrast, nomothetic span of general intelligence tests is often strong but construct representation remains a continuous academic discourse [54]. Therefore, the generated number series items must be evaluated for aspects of nomothetic span, allowing specific predictions about external correlates of scores to be made from reference tests measuring similar constructs. The 16-item International Cognitive Ability Resource (ICAR) short form test [55] was used as an external measure of the nomothetic span. A four-factor solution was previously identified to be the optimal solution with different item types representing different factors [55]. With these goals in mind, this study examines the item properties to better understand the shared variance of the item difficulties and cognitive operators, as well as the construct and criterion validity of the newly developed automatic number series item generator.

## 2. Method

### 2.1. Measures

The ANSIG was created using the R statistical programming language [56]. Thirteen item models were developed based on the five cognitive operators. However, only item models three to thirteen were used in this study, as the first two were deemed too easy for the adult population. Five items were randomly selected to represent each item model, resulting in a total of 55 items. The items were then split into two tests (Form A and Form B) to reduce the total number of items each participant had to complete. Five items from both item models three and eight were used as anchor items to bridge the scores between both test forms. Items from item models three, four, six, eight, ten and twelve were in Form A (*n* = 30 items). Items from item models three, five, seven, eight, nine, eleven and thirteen were in Form B (*n* = 35 items). The study was conducted online using an open source testing platform (Concerto v5: [57]). The items were presented without time limitation and participants were not allowed to return to the preceding item after moving onto the next one. The Person Separation index was used to evaluate the reliability of the number series tests. It is a reliability statistic comparable to Cronbach’s alpha [58]. Person Separation index of values >0.70 indicates adequate reliability [59].

The 16-item ICAR short form test [55] was administered after participants had completed the number series items, serving as an external measure of the nomothetic span. This instrument is a public-domain measure with four subscales: Matrix Reasoning, 3D Rotation, Letter-Number Series and Verbal Reasoning. Previous report provided evidence of adequate convergent validity with several widely accepted measures of cognitive ability [55]. Cronbach’s alpha [58] was used to evaluate the internal consistency of the test. sIn the current study, the scale showed adequate internal consistency (Cronbach’s α = 0.68, omega total ω = 0.70).

### 2.2. Participants

Participants were recruited via a crowd-sourcing website: Amazon’s TurkPrime (https://www.turkprime.com). TurkPrime is designed as an online research platform to support experimental tasks and conduct comprehensive surveys that are common to psychological sciences. [60,61]. It is increasingly being used to recruit more representative and diverse samples other than undergraduate students [62,63,64].

Participants were first presented with the number series items and then asked to fill in their demographic information (Table 3). The test data was collected online based on convenience sampling, where participants were randomly assigned to either Test Form A or Test Form B. Thus, participants were treated as equivalent. They were subsequently asked to complete the 16-item ICAR short form test. 396 participants completed Form A and 174 participants completed Form B. Two participants from each form chose not to reveal their demographic information. Among the 570 participants who completed the number series test, 390 participants also completed the 16-item ICAR short form test.

### 2.3. Data Analyses

The items from both forms were concurrently calibrated with a Rasch model using ten common items as an anchor. As such, items not taken by a particular group were treated as missing by design [65]. Conditional maximum likelihood estimation [66] was used to estimate item difficulty parameters, placing estimates for both forms onto a common metric. We used R programming language for the analysis. Specifically, we employed the R package eRm [67] to estimate the item difficulties of the Rasch model and the R package lme4 [68] to estimate the item difficulties of the LLTM and LLTM plus error [69]. The maximum likelihood estimation method used by the lme4 R package is based on the Laplace approximation of the likelihood [70], that is, an approximation to the likelihood is maximized. However, the Laplace approximation is reasonably accurate in applications such as the current one [69].

The Rasch model for dichotomous data is expressed in the following way:

(1) $$P\left(x_{vi}=1|\theta_{v},\delta_{i}\right)=\frac{exp\left(\theta_{v}-\delta_{i}\right)}{1+exp\left(\theta_{v}-\delta_{i}\right)}$$

where the probability *P* that the *v*th person solves the *i*th item correctly is conditional on the difference between the person parameter ($\theta_{v}$) and the difficulty of that item ($\delta_{i}$). A Wald test was subsequently used to compare item difficulty estimates of the two forms [71]. The sample was split into subsamples and the item difficulty parameter was estimated for each item in each subsample. The statistical hypothesis is that the item difficulty estimates are the same across subsamples. Items that were significantly different between sub-samples were removed. Andersen’s Likelihood Ratio (LR) test [72] was used to assess the overall Rasch model fit, which compares the item parameter estimates of sub-samples (mean-split) to the entire sample [73].

As the generator was created based on a cognitive model, the Linear Logistic Test Model (LLTM [49]) and its descendants were used to evaluate the extent to which the cognitive operators predict item difficulty. The LLTM assumes that the item parameters can be decomposed into a weighted sum of elementary parameters, allowing the item difficulty to be conceived as a function of hypothesised cognitive operations that are involved in the solution process [74]. LLTM is often considered as a restricted form of the Rasch model, with the assumption that the item difficulty parameter is a linear combination of several hypothesised elementary parameters [75]:

(2) $$\delta_{i}=\overset{m}{\underset{j=1}{\sum }}q_{ij}\eta_{j}$$

where the $q_{ij}$ (*j* = 1,2, …, *m*) are coefficients for the elementary components, $\eta_{j}$ are the hypothetical frequencies with which each component *j* influences the solution of each item *i*. The Q-matrix (Table 4) acts as a covariate matrix during estimation and is converted into a long-form matrix. This is subsequently known as the item property matrix, which is needed for the LLTM to explain the item easiness (difficulty) in terms of elementary components. The LLTM does not include an error term and therefore, assumes that the variance in item difficulty can be fully explained by the elementary components. However, it is common for Rasch item difficulty parameters to deviate from the item difficulty parameters predicted under the LLTM. This often leads to the goodness of fit of the LLTM to be inferior to that of the Rasch model. Thus, a further extension to the LLTM was made by Janssen et al. [50], who added a random error term to the function:

(3) $$\delta_{i}=\overset{m}{\underset{j=1}{\sum }}q_{ij}\eta_{j}+\varepsilon_{i}$$

where the error term $\varepsilon_{i}$ takes into account the residual variance of item difficulty that is not explained by the LLTM, so that the model need not explain the item difficulty estimates perfectly. The inclusion of an error term into the LLTM will give items both a structural part that is described by the linear predictors and an item-specific deviation part that is described by the error term. The smaller the residual variance the better the explanatory power of the item predictors. The person’s ability parameter is estimated by treating it as a random effect under the Generalised Linear Mixed Model [69]. The purpose of the random error term is to define a random residual for both items and persons with responses nested within items and persons, respectively [76].

We hypothesised that five cognitive operators were employed to solve the items: (1) Apprehension of succession, (2) Parallel sequences, (3) Cluster Formation, (4) Non-progressive coefficient patterns and (5) Complex progressive coefficient patterns. A design-matrix was created to account for the thirteen item models that resulted from these five cognitive operators. Table 4 shows the Q-matrix for the item models that were used in this study. Model selection was based upon two goodness-of-fit indices: the Akaike information criterion (AIC; [77]) and the Bayesian information criterion (BIC; [78]). The AIC selects the model that minimises the mean squared error of prediction, while the BIC, following certain assumptions, selects the true model given that it is a candidate among other models [79].

A Q-matrix (Table 5) relating to the four cognitive operators proposed by Holzman et al. [23] was also created to compare the item difficulty parameters estimated using the LLTM. We hypothesised that the correlation between both models should be similar to each other, since the proposed cognitive operators in this research are mapped onto those proposed by Holzman et al. [23]. Moreover, we hypothesised that the proposed cognitive operators in this research are more informative in explaining the between-item family variance as it provides a more transparent approach to item generation, thus providing a better model fit to the data. More information regarding the identification and specification of Q-matrix may be found elsewhere [80,81].

The 16-item ICAR short form test was employed to evaluate the nomothetic span of the number series items. The ICAR items were subset based on item types and a pairwise Pearson product-moment correlation analysis was conducted to investigate the relationship of the person-level *θ* estimates with the different cognitive domains of the 16-item ICAR short form test.

## 3. Results

### 3.1. Rasch Model Fit

In the Wald-Type test analysis, six items displayed significant differences between sub-samples. Two items were from item model four, one item from item model six, one item from item model eight and two items from item model twelve. These items showed statistical rejections at 5% for the Wald-type test and thus, contributed to overall test misfit. They were subsequently removed from the analysis. Of the six items that were removed, only one was found in both forms, resulting in nine instead of ten common items. The Wald-Type test was repeated on the remaining 49 items and the result indicated that the item difficulty estimates did not differ significantly between sub-samples.

**Figure 1:**
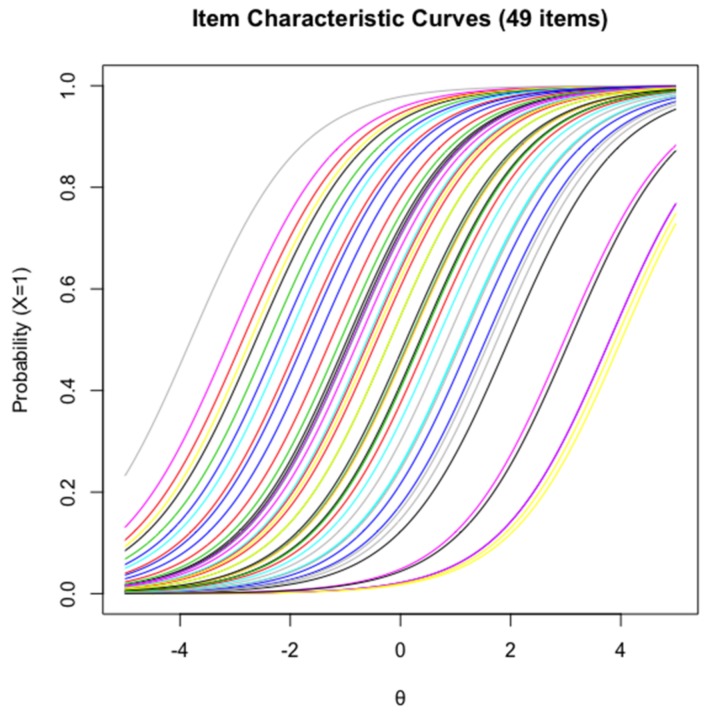
Item Characteristic curves for Form A and Form B.

The LR [72] test results showed that the assumption of item homogeneity was not violated (*χ*^2^(48) = 45.83, *p* = 0.56), suggesting that the remaining item models fitted the Rasch model well. Items covered a wide range of difficulty parameter, which in a logit scale, goes from −3.80 (easiest item) to 4.01 (most difficulty item). This can be seen in the ICC plot with regard to their horizontal locations ranging from theta −4 to 4 in Figure 1 respectively, with each coloured sigmoid line representing an item at different levels of ability.

### 3.2. Item and Item Model Variation in Difficulty

**Figure 2:**
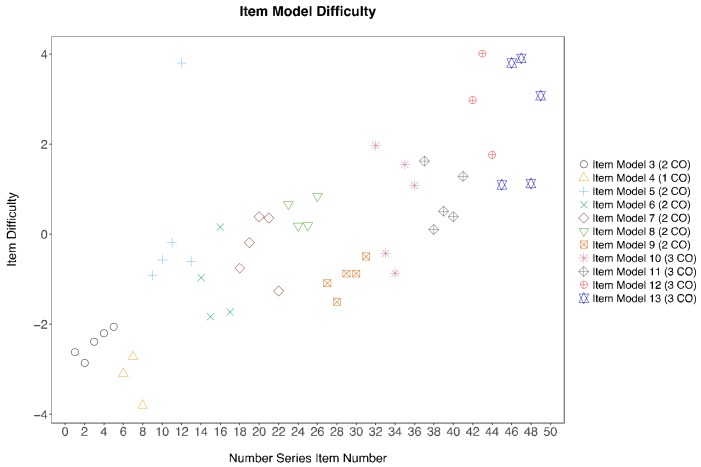
Item difficulty variations between and within item models using Rasch estimates. CO = cognitive operators.

Recall that the item difficulty level is hypothesised to be attributed to the demand of different cognitive operators employed to solve the items. Examining the item difficulty estimated from the Rasch Model, difficulties of items generated from the same item model tend to cluster, although outliers do occur (Figure 2). Namely, one item from item model five was estimated to have a much higher item difficulty level than the other items within the same model. One item from item model twelve and two items from item model thirteen were also found to have much lower item difficulties than the other five items. Note that the level of item difficulties is variable among those using the same number of cognitive operators. Despite holding the same number of cognitive rules in the working memory, variations in the cognitive operators employed to solve the problem led to different amount of information being held in the working memory. This resulted in differences in the item difficulty level. Except for item model 4, where only one cognitive operator is involved, there is a general upward trend in item difficulty from item model 3 to item model 13.

### 3.3. Test Information

**Figure 3:**
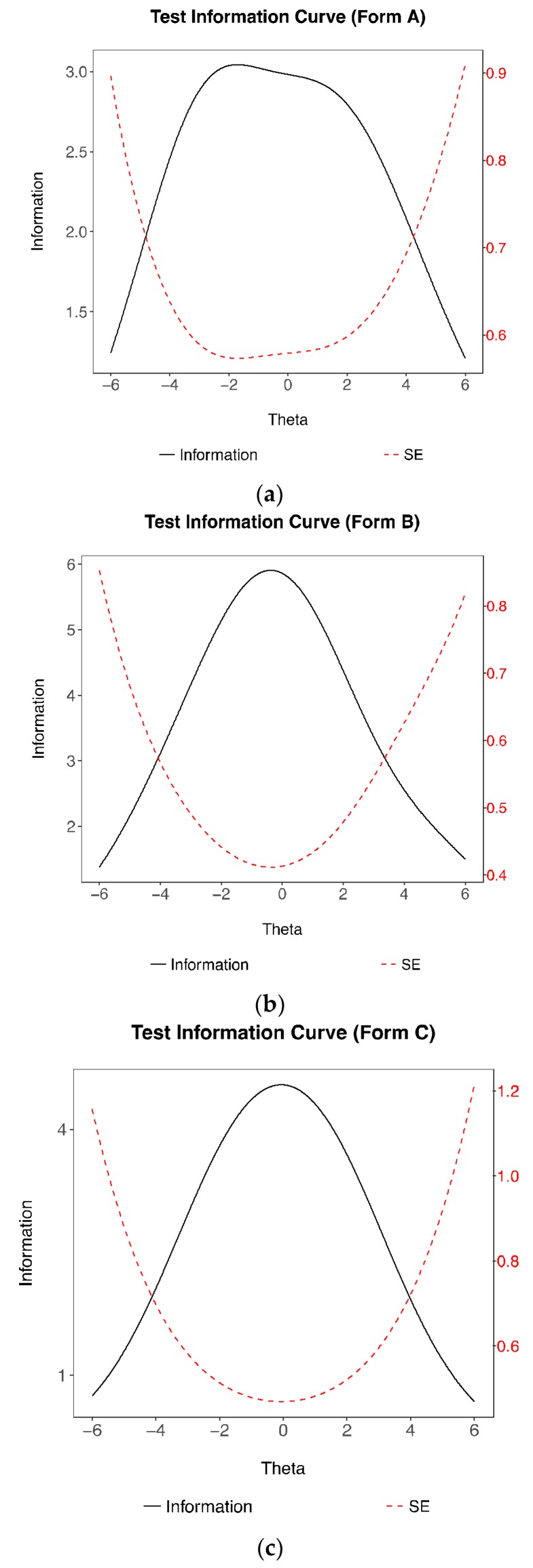
Test Information Curves for (**a**) Form A, (**b**) Form B and (**c**) Form C.

As none of the participants in this study completed all 49 items, the test information is reported separately: The maximum test information for Form A (*n* = 23 items) is 3.04 and 5.91 for Form B (*n* = 34 items) with a person separation reliability of 0.85 and 0.89, respectively. The test information for Form A is relatively flat between the theta range of −2 to 2, whereas the maximum information for Form B is given at *θ* = −0.2 (Figure 3). Both tests show a downward trend in the amount of information as it moves towards the lower end of the ability scale. Under IRT modelling, a scale can have uneven information functions based on at least two reasons: (a) the item difficulty estimates are clustered together in the trait continuum. For example, item difficulty estimates that are grouped on the higher end of the trait continuum will result in the scale yielding more precise measurement for individuals with higher trait values than for individuals with moderate to lower trait values. (b) The item discrimination values are differentially concentrated in certain regions of the trait range. The latter reason is of no concern to this research because the discrimination parameter is fixed in the Rasch model. As shown in Figure 3, the test information plot for Form A informs us that the items have a wide range of item difficulty estimates, while Form B consist of items grouped together with average difficulty.

An alternative version of the test (Form C) instead of a common scale was created using items taken from Form A and Form B to reduce test information inflation. The test information curve was created solely based on the existing psychometric data. From the set of forty-nine items, three items within an interval of item difficulty estimates of 0.5 ranging from approximately −2 to 2 were randomly drawn, resulting in 24 items. When the items from both forms are combined, the test information plot for Form C shows higher information levels than form A and a wider curve, albeit lower test information, around the average ability than form B. The maximum test information of 4.54 is approximately located at theta = 0, which is equivalent to an internal consistency reliability of 0.78. Given these characteristics, the items in this study encapsulate a wide range of ability and are suggested to be adequately reliable.

### 3.4. LLTM(s) Comparison with Different Q-Matrices

The correlation between the item difficulty parameters estimated by the LLTM using the Q-matrix with the newly revised cognitive operators and those proposed by Holzman et al. [23] was *r* = 0.98 (*p* < 0.001), with the variance explained at *R*^2^ = 0.96. The models were nested within each other: This is not obvious from the Q-matrices but all columns of the Holzman et al. [23] Q-matrix are linear combinations of the columns in the newly revised Q-matrix (see Table 1). A likelihood ratio test for nested models (*χ*^2^(2) = 115.9, *p* < 0.0001) and information criteria indicated superior fit of the newly revised cognitive operators (AIC = 14,901.8 and BIC = 14,955.2 for newly revised vs. AIC = 15,013.7 and BIC = 15,051.9 for Holzman et al. [23].

### 3.5. Difficulty Prediction with the LLTM and LLTM Plus Error

All five cognitive operators contributed significantly to the prediction of the item easiness parameter under the LLTM (Table 6). The result suggests that four of the cognitive operators have an increasing impact on item difficulty. Aside from the AOS cognitive operator, which has an opposite effect on difficulty, the PCP cognitive operator was found to have the greatest impact, while the PS had the smallest impact on difficulty. Thus, one can assume that the average participants may have employed these cognitive operators while they were trying to solve the items.

Under the LLTM plus error, the AOS cognitive operator was however, found not to be significantly different from the baseline difficulty (Table 6). The LLTM plus error fitted significantly better than the LLTM (*χ*^2^(1) = 1444, *p* < 0.001). Since the discrepancies between the parameter estimates of all models were not substantial, the LLTM plus error is the preferred choice from a model comparison perspective.

### 3.6. Goodness of Fit between the Rasch, LLTM and LLTM Plus Error 

Table 7 reports the goodness of fit indices (AIC and BIC) for the three different models. Indices with lower values indicate a better fit. Given the results of the goodness of fit indices, the Rasch model and the LLTM plus error are considered better fitting models than the LLTM. The BIC index is the lowest for the LLTM plus error, while the Rasch model exhibited the lowest value for the AIC index.

### 3.7. Item and Person Parameter Estimates

Table 8 shows the Rasch, LLTM and LLTM plus error item difficulty parameter estimates and standard errors. The standard errors for the item parameters under the LLTM and LLTM plus error were derived from bootstrapping (*n iterations* = 1000) the models respectively.

The correlation between the item difficulty parameters estimated by the Rasch and both the LLTM(s) were *r* = 0.83 (*p* < 0.001), with the variance explained at *R*^2^ = 0.69. The results suggest that the LLTM(s) accounted for 69% of the total variance of the item difficulty parameters estimated by the Rasch model. Finally, the person parameter correlations between the models are reported in Table 9.

### 3.8. Nomothetic Span

Table 10 shows that number series scores were moderately correlated (Form A: *r* = 0.60, *p* < 0.001; Form B: *r* = 0.66, *p* < 0.001) with the 16-item ICAR short form test scores. However, given the low reliabilities of the four subscales (0.34 to 0.75) in the 16-item ICAR short form test, it would be useful to correct these correlations for attenuation. Assuming that the person separation reliability of the number series is 0.85 for Form A and 0.89 for Form B, the correlation between the 16-item ICAR short form test and number series increases to 0.79 (*p* < 0.001) and 0.84 *(p* < 0.001) respectively. That is, after correction for attenuation, Form A shares 62% and Form B shares 71% of the variance with the 16-item ICAR short form test. This implies even stronger validity for the test. Being related to but distinct from the ICAR scales, provides evidence that the test brings added value to measures of general cognitive ability. This indicates that the items exhibited nomothetic span, as displayed with the significant correlations with the four item types.

## 4. Discussion

This paper successfully developed an original cognitive model for number series items. In order to predict item model difficulty, we proposed five cognitive operators that weight differently on the cognitive determinants proposed by Holzman et al. [23]. Based on these five cognitive operators, thirteen item models were developed and eleven investigated in the current paper. Five items were randomly drawn from each item model, resulting in fifty-five items. Findings supported that the item difficulty estimates that use the cognitive operators proposed by Holzman et al. [23] and the ones proposed in this study are similar. Additionally, the LLTM using the newly revised cognitive operators was found to be a significantly better fit to the data than that of the LLTM using the Holzman et al. [23] cognitive operators. Subsequent LLTM analyses used the revised cognitive operators for the Q-matrix. Results revealed that the item difficulty parameters estimated by the LLTM(s) had substantial correlations with those estimated by the Rasch model. All five cognitive operators prescribed in the Q-matrix were significant predictors in the LLTM, while the LLTM plus error had four significant cognitive operators. The inclusion of a random error term allows the model to account for the variance in item difficulty not explained by the cognitive operators. Model fit selection established that the LLTM plus error fits significantly better than the LLTM. Additionally, the automatically generated number series items exhibited adequate nomothetic span based on the correlation analysis with the 16-item ICAR short form test and its individual item types. Establishing both construct representation and nomothetic span indicates that the final automatically generated number series item set achieved construct validation.

Only the use of the AOS cognitive operator was not significantly different from the baseline difficulty under the LLTM plus error. A potential explanation may be that the rule is too easily detectable and hence, did not interfere with the solution process. This is not surprising, as it serves as a baseline operator and only requires participants to identify that the logic to the correct answer is solely based on the simple linear sequence of the number series. Nevertheless, the lack of impact shows that it is a promising candidate for constructing superficial variations (i.e., numerical values) in surface information in order to reduce item recognition effects.

PS had the least impact on item difficulty compared to CF, NPCP and PCP, which was expected because it only requires a small demand on the participants’ cognitive ability to discover the cycle of the numeric patterns. However, it did have a higher impact on item difficulty than the AOS, indicating more demand on discovery of periodicity. Identification of CF required high level of relation detection and had relatively higher impact on item difficulty than PS. NPCP, followed by PCP, displayed the highest impact on item difficulty, confirming the hypothesis that these 2 cognitive operators posed the highest demand on pattern description and extrapolation compared to the other cognitive operators. Thus, the variance in the influence of cognitive operators on the item difficulty provides clear evidence for the mapping relationship between the cognitive operators and the cognitive determinants proposed by Holzman et al. [23] as presented in Table 1. More importantly, it shows that the newly proposed cognitive operators lend systematic guidance on future test development.

There are variations of item difficulty both between and within item models. The results showed that some item models (e.g., item model 12) had more prominent within-variation than others. While the between-model variation is explained by the LLTM, the within-model variation is less understood. A potential explanation for differences in the level of within-model variations is due to processes in identifying the relations between the number series items. LeFevre and Bisanz [24] suggested that the recognition of memorised sequences, calculation and checking were procedures involved in identifying the relations between the numbers in the sequence. Memorised sequences (e.g., 5 10 15 20 25) and calculations are often easier and require less effortful processes to assist in verification. While two problems with the same cognitive operators may appear to demand equivalent effort in processing, familiarity in number sequences may result in easier and more accurate identification of a sequence when the memorial representation of that sequence is activated. On the other hand, less familiarity in the number sequences will require more effortful processing and lead to slower and less accurate identification of a sequence.

In the current study, the empirical methods employed were not able to account for variations within the item models. As the priority of the current research is to develop a generator based on a cognitive model, the goal was to figure out how cognitive processes might play a role in impacting item difficulty rather than evaluating potential reasons for variations in item difficulty within item families. Furthermore, only 5 items were generated from each item model, which is considered insufficient for studying within-family variations. Nevertheless, complex models are more likely to be successful as they may provide a more in-depth explanation of predicting item difficulty. In future research, techniques employing advance hierarchical modelling can be used to account for the dependencies among the items from the same item family [45,46,47]. For example, given that variations within item family were found, employing the additive multilevel item structure models with random residual [35] may allow us to examine the effect of within-model item difficulty variation in follow-up studies.

The rules we propose here are different from the ones proposed in previous literature. For example, the radicals proposed by Arendasy and Sommer [26], i.e. rule span, number of rules, rule complexity and periodicity, which focus more on the quantity of each feature in the number series questions, whereas the rules we used pay more attention to the cognitive processes employed in solving the questions. It is possible that combining both sets of rules will be able to explain more variance observed in the item difficulty. Future research is warranted to explore such possibility.

Participants’ mathematical reasoning ability was not included in the research, as we focused on understanding the extent to which individuals used the hypothesised cognitive operators to solve the problems rather than employing complex mathematical algorithms to achieve the correct answer. However, their mathematical reasoning ability could be another potential explanation for the differences found between the item difficulty estimates despite employing the same cognitive operators. For example, an item in item model 5 was considered more difficult than the other items (Figure 2), probably because that item had inconsistent rules between the sub-sequences (product of 3 and product of 2), whereas the rules were always consistent (product of 2) in the other items in the same item model. As in the case of item model 12, the simpler items had increments of less than 10, which is usually considered simpler arithmetic skill. Hence, the inclusion of more cognitive operators (i.e., mathematical reasoning ability) or increasing the weights of specific cognitive operators that uses more complex arithmetic operators within the Q-matrix may improve the item model difficulty estimation and provide new insights into the estimation of item model difficulty, bridging the gap of the LLTM estimates and the Rasch model.

The Rasch model uses the raw scores and response patterns to estimate item difficulties, providing a more accurate item difficulty estimates than the LLTM. This is evident by the overall comparison between the two models, where the observed data is found to fit the Rasch model significantly better than the LLTM (Table 7). Similar findings are common in the literature as the LLTM is comparable to a regression model that aims to explain total variance. However, the LLTM does not typically account for the total variance and hence, is more likely to be rejected [69]. Despite rejecting the LLTM, van der Linden and Hambleton [82] highlighted that the LLTM is still beneficial, because it allows one to predict item difficulty of new items approximately. Furthermore, the LLTM estimates are able to relate item performance to cognitive theory that has been proven useful in areas such as assessing treatment effects and modelling item bias [83].

The LLTM fit is improved by adding a homoscedastic error term. The inclusion of the error term accounts for the residual variance, allowing for an imperfect explanation to the variance of the item difficulty estimates. As shown in Table 7, the LLTM plus error fits the data significantly better than the LLTM. The LLTM plus error is considered as the most parsimonious model (with the lowest BIC) compared to the Rasch model and the LLTM, indicating that the internal structure of the items during the construction process is best delineated by the proposed cognitive operators in this study. The AIC on the other hand, tends to select models that are more saturated [84]. Thus, it is unsurprising that the Rasch model had the lowest AIC value, as a linear constraint is not imposed on the estimation of the item difficulty parameters. 

Nonetheless, the Q-matrix should include more parameters (i.e., rules) to identify possible sub-domains within an item model to increase the predictability of item difficulty for newly generated items. Furthermore, the Q-matrix in this research only consist of 8 different combinations instead of a maximum of 32 (2^5^) combinations. Thus, more item models should be included in future research that will result in greater variations in the number series items. Maximising the number of item models will provide researchers an opportunity to study interaction effects between the interrelated cognitive operators involved in the task solution and evaluate the structural relationships between the individual, the task and the result of their interaction.

In this research, six items from Form A were removed. Using methods such as automatic min-max [5] could help reduce the risk of differential item functioning and exclude construct-unrelated interfering variances by introducing functional constraints during the item design phase. For example, it is necessary to constrain the magnitude of the numbers in the generated individual sequences (e.g., 28 30 32) such that the values are not overly large (e.g., 3489 3491 3493), so that participants do not form an initial impression that the item is extremely difficult. Thus, the role of these constraints acts as a quality control component of the generator, ensuring that the automatically generated items present a valid construct representation. This study did not include the employment of functional constrains so that we could observe the variances within item models with the proposed notional system. This approach encourages the generative veracity of the AIG so that the strengths and weaknesses of the modelling procedures can be discerned and revised accordingly in future studies. Nevertheless, it is worthwhile to point out that the items employed in this study did not use numbers with exceptionally large values.

The present research attempts to define a bank of item models by means of constructing new rules following substantive cognitive theory. Our research on AIG has adopted an exploratory approach. By exploratory, we mean that item models were first identified using the hypothesised cognitive operators and followed by the generation of new items. This approach to item generation may be appropriate at the early phase but is considered inefficient when large number of items are created without limitations, because content classification is conducted only after the items are produced [85]. Enforcing functional constraints and further refinement of the Q-matrix is necessary to produce new items which need not be piloted empirically prior to administration. Nevertheless, the notational system provided in this paper (Table 2) encourages researchers to generate their own items based on the item models and use different empirical evaluation methods to study the item properties. It is worth mentioning that the automatic item generator developed in the study is fully available as an R package (“*numGen*” [1]). Detailed instruction is provided in the instructional manual. It is the hope of the authors that test developers can share more than results in future research and practice, to further advance the study in intelligence research and psychometrics.

Lastly, in the area of automatic item generators, the major benefit is to produce items that have predictable excellent psychometric properties. Thus, the automation of item construction with predictable item difficulties would offer new possibilities for computerised adaptive testing [86,87]. The achievement of this goal would thus require further revision of the cognitive framework and the evaluation of more advanced psychometric methods.

## Figures and Tables

**Table 1:** Description of cognitive operators.

Index	Cognitive Operator	Description	Example	Relation Detection	Discovery of Periodicity	Pattern Description	Extrapolation
1	Apprehension of succession	Identification of the missing value is determined by the immediate preceding value.	1,2,3,4,5,6	Low	Low	Low	Low
2	Identification of parallel sequences	Two parallel sequences are inherent within an item that forms two number series.	1,2,1,4,1,6		High		
3	Object cluster formation	The missing value is determined by the relationship within groups of elements.	1,1,1,2,2,2	High			
4	Non-progressive coefficient patterns	Identification of the missing value is influenced by the difference between two preceding values.	2,4,6,8,10			Middle	Middle
5	Complex progressive coefficient patterns	The missing value increases or decreases largely based on more than one arithmetic operation or increasing values.	1,2,4,7,11			High	High

**Table 2:** Item model description.

Item Model	Sample Item	Task Objective	Item Logic
1	10 20 30 40 (50)	Elementary understanding of sequence succession	Simple linear sequences which do not require use of advanced arithmetic operations, such as ordered multiples of 1, 10, or 100.
			*Example: A sequence of ordered multiples of 10.*
2	1 1 1 5 5 (5)	Understanding of object clusters	Sequences consist of elements belonging to two homogeneous groups with equal number of elements. Missing element belongs to the group with fewer elements present in the sequence.
			*Example: Ordered groups of 1s and 5s. Number 5 added to the sequence results in equal number of elements in the two groups.*
3	1 2 4 8 16 (32)	Use of basic algebraic skills	Each element in the sequence is derived from the preceding by applying one of four basic arithmetic operations—addition, subtraction, multiplication, or division. Coefficient of change is invariant across the sequence.
			*Example: A sequence of elements using a multiplication of 2.*
4	1 10 2 20 3 30 (4) (40)	Identification of co-occurring relationships between elements (minimum use of arithmetic skills)	Sequences that consist of regularly alternating parallel sub-sequences. Understanding of succession requires minimum use of algebraic skills. Sub-sequences involve items from item model 1.
			*Example: Odd elements of the sequence are multiples of 1 and even elements of the sequence are multiples of 10.*
5	2 7 4 14 8 28 16 (56) (32)	Identification of co-occurring relationships between elements (with use of arithmetic skills)	Logic analogous to the item model 4 but at least one sub-sequence involves the basic arithmetic operations. Sequences combine items from item models 1 and 3.
			*Example: Both odd and even elements of the sequence are multiplied by 2 but with different starting values.*
6	2 4 7 11 16 (22)	Identification of progressively evolving coefficients of change	Non-linear progressive sequences which require a higher level of abstraction; the coefficient of change between two neighbouring elements is not invariable and its elements form a new sequence. The coefficient sequences correspond to items from item models 1 and 3.
			*Example: The coefficient of change between each pair of neighbouring elements in the sequence increases by 1.*
7	3 10 24 52 108 (220)	Identification of complex coefficients of change	Ability to identify complex coefficients; the coefficient of change involves a combination of arithmetic operations (e.g., addition and multiplication) applied serially.
			*Example: Each element in the sequence is derived from the preceding by adding two and multiplying the result by two.*
8	1 3 8 10 207 (209)	Identification of non-successive relationships within a sequence	Sequences consist of pairs (or triads) of elements which share common features, while the values across pairs (triads) are unrelated.
			*Example: A sequence formed by three pairs of elements. The difference between elements in each pair equals two. Individual pairs are not otherwise related.*
9	1 1 2 3 5 8 (13)	Identification of relationships within a chain of elements	Progressive sequences which involve relationships between multiple preceding objects (e.g., Fibonacci sequence).
			*Example: Each element of the sequence is a result of addition of its two preceding elements.*
10	2 15 4 17 7 19 11 21 16 (23) (22)	Combined identification of parallel sub-sequences and progressively evolving coefficients of change	Logic analogous to the item model 4 but at least one sub-sequence involves a progressively evolving coefficient. Sub-sequences involve items from item models 1, 3 and 6.
			*Example: The coefficient of change between odd elements in the sequence increases by 1. The even elements increase by 2.*
11	1 7 14 20 40 46 (92) (98)	Identification of alternating coefficients of change	Progressively evolving sequences whose elements develop following multiple alternating rules (e.g., addition for even elements and multiplication for odd elements).
			*Example: A sequence whose coefficient of change alternates between (+6) and (×2).*
12	1 22 44 2 66 88 3 110 (132) (4)	Identification of unevenly ordered sub-sequences	Logic analogous to the item model 4 but sub-sequences follow irregular pattern: S_1_, S_2_, S_2_, S_1_, S_2_, S_2_, S_1_, S_2_, S_2_. Sub-sequences involve items from item models 1, 3 and 6.
			*Example: Sub-sequences with coefficients of (+1) and (+22) ordered according to the pattern above.*
13	1 5 8 3 209 212 5 41 (44) (7)	Combined identification of unevenly ordered sub-sequences and non-successive relationships between elements	Logic analogous to the item model 12 but the second sequence belongs to the item model 8. As a result, pairs of elements following certain rule are embedded into a progressive sequence.
			*Example: Sequence with coefficient of (+2) is interposed with pairs of elements which differ by 3.*

**Table 3:** Demographics comparison between Form A and Form B.

	Form A (*n* = 396)	Form B (*n* = 174)
Gender		
Male	124 (31.3%)	51 (29.3%)
Female	270 (68.2%)	121 (69.5%)
Prefer not to say	2 (0.5%)	2 (1.2%)
Nationality		
American	337 (85.1%)	146 (83.9%)
Others	57 (14.4%)	26 (14.9%)
Prefer not to say	2 (0.5%)	2 (1.2%)
Education		
Doctorate	9 (2.3%)	6 (3.5%)
Master’s Degree	67 (16.9%)	25 (14.4%)
Bachelor’s Degree	150 (37.9%)	67 (38.5%)
Vocational Qualifications	55 (13.9%)	32 (18.4%)
At least Primary Education	103 (26%)	42 (23.6%)
Prefer not to say	2 (0.5%)	2 (1.2%)

**Table 4:** Q-matrix of the proposed cognitive operators.

Item Model	Apprehension of Succession	Parallel Sequences	Cluster Formation	Non-Progressive Coefficient Patterns	Progressive Coefficient Patterns
1	1	0	0	0	0
2	0	0	1	0	0
3	1	0	0	1	0
4	0	1	0	0	0
5	0	1	0	1	0
6	1	0	0	0	1
7	1	0	0	0	1
8	0	0	1	1	0
9	1	0	1	1	0
10	0	1	0	0	1
11	1	0	1	1	0
12	0	1	1	1	0
13	0	1	1	1	0

**Table 5:** Q-matrix of the cognitive operators proposed by Holzman et al. [23].

Item Model	Relation Detection	Discovery of Periodicity	Pattern Description	Extrapolation
1	0	0	0	0
2	1	0	0	0
3	0	0	1	1
4	0	1	0	0
5	0	1	1	1
6	0	0	2	2
7	0	0	2	2
8	1	0	1	1
9	1	0	1	1
10	0	1	2	2
11	1	0	1	1
12	1	1	1	1
13	1	1	1	1

**Table 6:** Cognitive operator estimates in the linear logistic test model (LLTM) and LLTM + *ε* predicting item easiness.

Effects	Parameter	LLTM		LLTM + *ε*	
		Estimate	SE	Estimate	SE
Fixed effects	Constant	4.77 ***	0.16	5.32 ***	0.85
	AOS	0.35 ***	0.07	0.37	0.58
	PS	−1.53 ***	0.07	−1.83 **	0.59
	CF	−2.13 ***	0.06	−2.25 ***	0.41
	NPCP	−2.65 ***	0.15	−3.09 ***	0.71
	PCP	−3.76 ***	0.14	−4.28 ***	0.69
		LLTM		LLTM + *ε*	
		Variance	Std. Dev	Variance	Std. Dev
Random effects	*θ*j (persons)	1.19	1.09	1.67	1.29
	*ɛ*i (item)	-	-	1.03	1.01

Note: AOS—Apprehension of succession, PS—Parallel sequences, CF—Cluster Formation, NPCP—Non-progressive coefficient patterns and PCP—Progressive coefficient patterns. LLTM is the linear logistic test model. LLTM + *ε* is the linear logistic test model plus error term. SE—Standard Error. Std. Dev—Standard Deviation. Cognitive operator estimates are beta coefficient estimates in predicting item easiness estimates. *** *p* < 0.001, ** *p* < 0.01.

**Table 7:** Goodness of fit.

Model	No. of Parameters	AIC	BIC
Rasch	50	13,321	13,702
LLTM	7	14,902	14,955
LLTM + *ε*	8	13,460	13,521

Note: AIC—Akaike Information Criterion; BIC—Bayesian Information Criterion.

**Table 8:** Rasch, LLTM and LLTM + *ε* item difficulty parameter estimates and standard error.

Item	Item Model	Rasch Estimate	Std. Error	LLTM	Bootstrap SE	LLTM + *ε*	Bootstrap SE
1	3	−2.62	0.18	−2.46	0.07	−2.60	0.34
2	3	−2.86	0.19	−2.46	0.07	−2.60	0.34
3	3	−2.39	0.16	−2.46	0.07	−2.60	0.34
4	3	−2.20	0.16	−2.46	0.07	−2.60	0.34
5	3	−2.06	0.15	−2.46	0.07	−2.60	0.34
6	4	−3.10	0.24	−3.24	0.14	−3.49	0.58
7	4	−2.72	0.21	−3.24	0.14	−3.49	0.58
8	4	−3.80	0.31	−3.24	0.14	−3.49	0.58
9	5	−0.91	0.21	−0.59	0.07	−0.40	0.36
10	5	−0.57	0.20	−0.59	0.07	−0.40	0.36
11	5	−0.19	0.19	−0.59	0.07	−0.40	0.36
12	5	3.80	0.32	−0.59	0.07	−0.40	0.36
13	5	−0.61	0.20	−0.59	0.07	−0.40	0.36
14	6	−0.97	0.14	−1.36	0.06	−1.40	0.31
15	6	−1.83	0.17	−1.36	0.06	−1.40	0.31
16	6	0.16	0.13	−1.36	0.06	−1.40	0.31
17	6	−1.73	0.16	−1.36	0.06	−1.40	0.31
18	7	−0.76	0.21	−1.36	0.06	−1.40	0.31
19	7	−0.19	0.19	−1.36	0.06	−1.40	0.31
20	7	0.39	0.19	−1.36	0.06	−1.40	0.31
21	7	0.36	0.19	−1.36	0.06	−1.40	0.31
22	7	−1.26	0.22	−1.36	0.06	−1.40	0.31
23	8	0.67	0.11	0.01	0.06	0.01	0.50
24	8	0.18	0.11	0.01	0.06	0.01	0.50
25	8	0.19	0.11	0.01	0.06	0.01	0.50
26	8	0.85	0.11	0.01	0.06	0.01	0.50
27	9	−1.08	0.22	−0.33	0.07	−0.35	0.27
28	9	−1.50	0.24	−0.33	0.07	−0.35	0.27
29	9	−0.87	0.21	−0.33	0.07	−0.35	0.27
30	9	−0.87	0.21	−0.33	0.07	−0.35	0.27
31	9	−0.50	0.20	−0.33	0.07	−0.35	0.27
32	10	1.97	0.15	0.51	0.06	0.80	0.32
33	10	−0.42	0.13	0.51	0.06	0.80	0.32
34	10	−0.86	0.14	0.51	0.06	0.80	0.32
35	10	1.55	0.14	0.51	0.06	0.80	0.32
36	10	1.09	0.13	0.51	0.06	0.80	0.32
37	11	1.62	0.20	−0.33	0.07	−0.35	0.27
38	11	0.11	0.19	−0.33	0.07	−0.35	0.27
39	11	0.51	0.19	−0.33	0.07	−0.35	0.27
40	11	0.39	0.19	−0.33	0.07	−0.35	0.27
41	11	1.29	0.19	−0.33	0.07	−0.35	0.27
42	12	2.98	0.18	1.54	0.07	1.84	0.29
43	12	4.01	0.25	1.54	0.07	1.84	0.29
44	12	1.76	0.14	1.54	0.07	1.84	0.29
45	13	1.10	0.19	1.54	0.07	1.84	0.29
46	13	3.80	0.32	1.54	0.07	1.84	0.29
47	13	3.91	0.33	1.54	0.07	1.84	0.29
48	13	1.13	0.19	1.54	0.07	1.84	0.29
49	13	3.08	0.26	1.54	0.07	1.84	0.29

Note: LLTM—Linear Logistic Test Model; LLTM + *ε* —Linear Logistic Test Model plus error term; SE—Standard Error.

**Table 9:** Person parameter correlations between models.

Models	Rasch	LLTM	LLTM + *ε*
Rasch	1	-	-
LLTM	0.998	1	-
LLTM + *ε*	1	0.998	1

Note: LLTM—Linear Logistic Test Model; LLTM + *ε* —Linear Logistic Test Model plus error.

**Table 10:** Correlations between the factor scores of the number series items and the 16-item International Cognitive Ability Resource short form overall and individual item types.

Variable	Numeric Series Ability (Form A)	Form A (Adjusted)	Numeric Series Ability (Form B)	Form B (Adjusted)
16-item ICAR Short Form Test	0.60 ***	0.79 ***	0.66 **	0.84 ***
Verbal Reasoning (4 items)	0.36 ***	0.56 ***	0.34 ***	0.62 ***
Letter-Number (4 items)	0.42 ***	0.64 ***	0.41 ***	0.58 ***
3D Rotation (4 items)	0.33 ***	0.46 ***	0.45 ***	0.55 ***
Matrix Reasoning (4 items)	0.40 ***	0.63 ***	0.28 **	0.49 ***

** Correlation is significant at the 0.01 level (2-tailed). *** Correlation is significant at the 0.001 level (2-tailed). Person separation reliability index is used to correct for correlations. Form A; *n* = 284. Form B; *n* = 106.
